# Evaluation of the repeatability and reliability of the cross-training specific Fight Gone Bad workout and its relation to aerobic fitness

**DOI:** 10.1038/s41598-021-86660-x

**Published:** 2021-03-31

**Authors:** Krzysztof Durkalec-Michalski, Emilia E. Zawieja, Bogna E. Zawieja, Tomasz Podgórski

**Affiliations:** 1 Department of Sports Dietetics, Poznan University of Physical Education, 61-871 Poznan, Poland; 2 Department of Human Nutrition and Dietetics, Poznan University of Life Sciences, 60-624 Poznan, Poland; 3 Department of Mathematical and Statistical Methods, Poznan University of Life Sciences, 60-637 Poznan, Poland; 4 Department of Physiology and Biochemistry, Poznan University of Physical Education, 61-871 Poznan, Poland

**Keywords:** Physiology, Biomarkers

## Abstract

Cross-training is a high-intensity functional training (HIFT) with multiple workout modalities. Despite the increasing number of studies in HIFT, there is still no validated test to measure its specific performance. It would also be advisable to determine whether selected cross-training workouts can implement a stimulus corresponding to maximize aerobic work. For these reasons, the purpose of our study was to evaluate the repeatability and reliability of Fight Gone Bad (FGB) workout and to assess its relationship with aerobic fitness. Twenty-one cross-training participants (9 females) finished the study protocol which included three two-day measurement sessions separated by 10 days. During each session, participants had their body composition measured, and they performed two exercise tests. The first test was an incremental cycling test to measure aerobic fitness, and the second was a cross-training specific FGB workout performed the next day. Reliability and repeatability were calculated from the three measurements. The total FGB Score (FGB_TOTAL_) showed excellent reliability (ICC 0.9, SEM 6%). Moreover, FGB_TOTAL_ was strongly correlated with aerobic fitness (i.e., time to exhaustion (T_exh_, R^2^ = 0.72), maximal workload (W_max_, R^2^ = 0.69), time to gas exchange threshold (T_GET_, R^2^ = 0.68), and peak oxygen uptake (VO_2peak_, R^2^ = 0.59). We also found that agreement between standardized FGB and standardized aerobic performance indices such as T_exh_, VO_2peak_, W_max_, maximum heart rate, T_GET_, and workload at gas exchange threshold was high by the Bland–Altman method. In conclusion, FGB is a reliable test that can be used in order to measure changes in cross-training performance caused by an intervention. Moreover, FGB is strongly correlated to aerobic fitness.

## Introduction

Cross-training has incredibly grown in popularity for over a dozen years. It combines ten key physical parameters to improve overall fitness: cardiovascular/respiratory endurance, stamina, strength, flexibility, power, speed, coordination, agility, balance, and accuracy^1–3^. Cross-training can be described as high-intensity functional training (HIFT) that incorporates exercises with barbells, kettlebells, dumbbells, cardiovascular tasks (i.e., running, rowing), and gymnastics (i.e., pull ups, muscle ups)^4^. It is varied in its nature, which appeals strongly to many people around the world.

In a systematic review, Haddock et al.^5^ suggests that HIFT increases aerobic fitness, strength, musculature, and endurance. Cosgrove et al.^6^ studied the influence of six months of HIFT on multiple fitness characteristics. The participants completed three separate days of assessments across 10 fitness domains before and after participating in the program for 6 months. They found that HIFT improved several fitness parameters, including flexibility, power, muscular endurance, and strength^6^. Murawska-Cialowicz et al.^7^ also showed that there was an improvement in aerobic fitness after 3 months of HIFT training in women but not in men. However, no changes in Wingate anaerobic power were observed.

Other investigators focused on finding the connection between HIFT performance and aerobic and anaerobic fitness as well as strength capabilities^8–11^. Butcher et al.^9^ studied whether physiological measures can predict selected cross-training benchmark performance. Aerobic fitness was measured as maximum oxygen uptake (VO_2max_) in a treadmill ramp test^9^. Anaerobic power was measured in a classical Wingate test on a cycloergometer. Cross-training performance was evaluated in benchmark workouts; ‘Fran’ (three rounds of thrusters (a thruster is a combination of a front squat and an overhead press) and pull-ups for 21, 15, and 9 repetitions), ‘Cindy’ (20 min of rounds of five pull-ups, ten push-ups, and 15 bodyweight squats), ‘Grace’ (30 clean and jerks for time) and ‘CrossFit Total’ (1 repetition maximum (1RM) back squat, overhead press, and deadlift). They found no correlation between HIFT performance and aerobic fitness. Bellar et al.^8^ aimed to investigate the relationship of aerobic fitness and anaerobic power with performance in two representative cross-training workouts. The first was 12 min (as many rounds as possible of 12 medicine ball throws, 12 kettlebell swings, and 12 burpee pullups), and the second was based on the total time to complete the prescribed exercise (3 rounds of 21–15–9 repetitions of a sumo deadlift high pull, box jumps, and farmer walk with bumper plates) in cross-training experienced and naïve adults^8^. The cross-training experienced group performed significantly better in both performance tests. In the experienced group, both cross-training tests results were correlated with VO_2max_, but in the naïve group, only the 21–15–9 test. Dexheimer et al.^10^ conducted a study aimed to determine which physiological performance measure could serve as the greatest indicator of cross-training workout performance. Participants completed a graded exercise test on a treadmill to determine VO_2max_, a 3-min all-out test, a Wingate test, ‘CrossFit Total’, and three HIFT benchmark trainings: ‘Grace’, ‘Fran’, and ‘Nancy’ (5 rounds of a 400 m run and 15 overhead squats). In comparison to Butcher and colleagues^9^, they found that ‘Fran’, ‘Nancy’, and ‘CrossFit Total’ were significantly correlated with VO_2max_, but only the score in ‘Nancy’ and ‘CrossFit Total’ were correlated with peak and average anaerobic power^10^. Interestingly, they showed that critical speed and total body strength are not crucial predictors of HIFT performance. Finally, Martínez-Gómez et al.^11^ hypothesized that a full squat might be used as a predictor of cross-training performance, at least for exercises involving lower-limb muscles. The participants performed a squat test to measure 1RM and five cross-training workouts from the CrossFit Opens in 2019. In the first workout of the day (WOD_1_), participants had to perform 225 repetitions of dumbbell snatches and burpee box jump-overs for time. In WOD_2_, the athletes had to perform as many repetitions as possible of 50-feet (15.24 m) weighted walking lunges, toes-to-bar, bar muscle ups, and power cleans in 12 min. In WOD_3_, contestants had to perform the maximum possible number of repeated circuits, each including chest-to-bar pull-ups and squat snatches (with weight progressively increasing from 95 to 265 lbs (43–120 kg)) in 8 min. In WOD_4_, participants had to perform as many repetitions as possible of deadlifts (225 lb (102 kg)), wall-ball shots (20 lb (9 kg) to a 10-feet (3 m) high target), rowing, and handstand push-ups in 13 min. In WOD_5_, participants had to perform 440 repetitions of thrusters (95 lb (45 kg)) and double-unders in the shortest time possible. Cross-training performance was then calculated from all five WODs. They found moderate to strong correlations between squat variables and all WODs^11^. The same for cross-training performance.

Because HIFT is becoming more and more popular every year, many studies are carried out to measure the influence of different dietary regimens and supplementation on cross-training performance^12,13^. However, it is difficult to compare those results, because authors use different cross-training tests. The reason for this is that there are no validated and well-described tests of cross-training performance. In the present paper, we proposed one of the cross-training benchmark workouts Fight Gone Bad (FGB) as a discipline-specific test to measure HIFT performance. FGB comprises 3 rounds of 5 different cross-training specific exercises, so there are 15 min of exercises with two 1-min breaks between the 1st and 2nd, and 2nd, and 3rd rounds. It requires most of the HIFT key physical traits such as power, strength, speed/strength endurance, balance, and stamina^1,2^. In addition, it can be performed by both experienced and inexperienced athletes, which makes it useful to track progress over time. That is why the purpose of this study is to evaluate the repeatability and reliability of FGB over repeated measurements and to assess its relation to aerobic performance. The secondary aim was to assess changes in hematological and biochemical parameters caused by FGB in order to have better insight into the organism’s response to such a workout.

## Results

### Body composition

There were no significant differences in body mass and composition between the three measurements (baseline and after 10 and 20 days), Table 1. Relative reliability (interclass correlation coefficient) was excellent (ICC > 0.9) for all measures by Bod Pod and FFM and body water (kg) by BIA. ICC was good for FM (kg and %) by BIA and low for body water (%). For Bod Pod, body mass, and FFM, the SEM was 1%, and it was 6% for FM (both in kg and %). For BIA, FFM SEM was 1%, for body water (kg) 5%, 10% for body water (%), 11% for FM (kg) and 12% for FM (%). For all Bod Pod measurements, SEM was less than SWC, indicating good ability of the test to detect small and meaningful changes. In BIA, SEM < SWC was observed only for FFM (kg) and body water (kg).

**Table 1:** Repeatability of body composition measurements.

	T_1_	T_2_	T_3_	*P* value ANOVA	ICC	*p*	SEM	SWC	MDC
**Bod Pod**									
Body weight (kg)	73.0	73.2	73.1	0.9994	1.00	< .0001	0.7	4.7	1.5
Fat mass (kg)	13.8	13.8	13.3	0.9421	0.97	< .0001	0.8	1.7	1.6
Fat-free mass (kg)	59.2	59.4	59.8	0.9915	1.00	< .0001	0.8	4.7	1.6
Fat mass (%)	19.3	19.3	18.7	0.9511	0.98	< .0001	1.1	2.4	2.2
**BIA**									
Fat mass (kg)	11.7	11.4	11.7	0.9553	0.89	< .0001	1.3	1.2	2.5
Fat-free mass (kg)	61.3	61.7	61.5	0.9953	0.99	< .0001	1.6	4.6	3.1
Fat mass (%)	16.4	16.0	16.4	0.9641	0.87	< .0001	2.0	1.8	3.9
Body water (kg)	44.5	44.4	44.3	0.9979	0.96	< .0001	2.0	3.3	4.0
Body water (%)	58.0	60.6	60.3	0.4066	0.29	< .0001	5.7	1.7	11.4

BIA, bioelectrical impedance analysis; ICC, interclass correlation coefficient; MDC, minimal detectable change; SEM, standard error of measurement; SWC, smallest worthwhile change.

### Fight Gone Bad performance

There were no significant differences in FGB performance between the three measurements (Table 2). ICC was excellent for FGB Round-1 (FGB_R-1_) and FGB_TOTAL_ and good for FGB Round-2 (FGB_R-2_) and FGB Round-3 (FGB_R-3_). SEM was 6, 8, 9 and 6% for FGB_R-1_, FGB_R-2_, FGB_R-3_, and FGB_TOTAL_, respectively. SEM was lower than SWC for FGB_R-1_ and FGB_TOTAL_, indicating good ability of the test to detect small and meaningful changes. However, for FGB_R-2_ and FGB_R-3_, SEM was higher than SWC. MDC values are given in Table 2.

**Table 2:** Repeatability of Fight Gone Bad performance.

	T_1_	T_2_	T_3_	*P*-value ANOVA	ICC	*p*	SEM	SWC	MDC
FGB_R-1_ (reps)	104 ± 22	105 ± 18	111 ± 19	0.4614	0.91	< .0001	6.4	7.0	12.6
FGB_R-2_ (reps)	85 ± 19	92 ± 16	91 ± 16	0.3295	0.84	< .0001	7.1	5.8	14.1
FGB_R-3_ (reps)	81 ± 17	87 ± 14	89 ± 15	0.2033	0.78	< .0001	7.7	5.1	15.1
FGB_TOTAL_ (reps)	270 ± 57	284 ± 48	291 ± 48	0.3644	0.90	< .0001	17.3	17.7	34.3

FGB_R-1_, Fight Gone Bad Round 1; FGB_R-2_, Fight Gone Bad Round 2; FGB_R-3_, Fight Gone Bad Round 3; FGB_TOTAL_, Fight Gone Bad total number of repetitions; ICC, interclass correlation coefficient; MDC, minimal detectable change; SEM, standard error of measurement; SWC, smallest worthwhile change.

There were no significant differences in HR during FGB between the three measurements (Table 3). ICC was good for FGB_R-2_, Rest_2_, Mean_15min_, and Mean_17min_. ICC was acceptable for Rest_1_ and FGB_R-3_ and low for FGB_R-1_. SEMs were low, between 2 and 5%. SEM was higher than SWC for all HR measurements in FGB. MDC values are given in Table 3.

**Table 3:** Repeatability of heart rate during Fight Gone Bad.

	T_1_	T_2_	T_3_	*P*-value ANOVA	ICC	*p*	SEM	SWC	MDC
FGB_R-1_ (bpm)	174 ± 9	165 ± 12	169 ± 14	0.0823	0.22	0.0970	8.3	2.2	16.4
Rest_1_ (bpm)	165 ± 13	159 ± 10	160 ± 13	0.2600	0.66	< .0001	5.5	2.8	10.9
FGB_R-2_ (bpm)	174 ± 10	170 ± 11	172 ± 14	0.4928	0.87	< .0001	3.3	3.1	6.6
Rest_2_ (bpm)	167 ± 13	163 ± 11	165 ± 11	0.5790	0.73	< .0001	4.7	2.8	9.2
FGB_R-3_ (bpm)	173 ± 10	171 ± 9	173 ± 11	0.8607	0.50	0.0040	3.0	1.2	5.9
Mean_15min_ (bpm)	173 ± 10	169 ± 11	172 ± 14	0.3549	0.79	< .0001	4.4	3.1	8.8
Mean_17min_ (bpm)	173 ± 11	168 ± 11	171 ± 13	0.3146	0.82	< .0001	4.0	3.0	8.0

FGB_R-1_, Fight Gone Bad Round 1; FGB_R-2_, Fight Gone Bad Round 2; FGB_R-3_, Fight Gone Bad Round 3; Mean_15min_, mean of FGB_R-1_, FGB_R-2_ and FGB_R-3_; ICC, interclass correlation coefficient; MDC, minimal detectable change; Mean_15min_, mean of FGB_TOTAL_; SEM, standard error of measurement; Rest_1_, rest period between round 1 and 2; Rest_2_, rest period between round 2 and 3; SWC, smallest worthwhile change.

### Aerobic fitness

Furthermore, there were no significant differences in ICT (Table 4). Relative reliability (interclass correlation coefficient) was excellent for T_exh_, W_max_, T_GET_, W_GET_, VO_2peak_, VCO_2peak_, and EE, good for HR_GET_ and VO_2GET_, and acceptable for HR_max_. SEM was low (< 5.99%) for HR_max_, HR_GET_, and VO_2peak_, and moderate (6–10%) for T_exh_, W_max_, T_GET_, W_GET_, and VO_2GET_. SEM for EE was 11%. SEM < SWC was observed for T_exh_, W_max_, T_GET_, W_GET_, VO_2peak_, VCO_2peak_, and EE. However, for HR_max_, HR_GET_, and VO_2GET_ SEM was higher than SWC.

**Table 4:** Repeatability of aerobic variables measured in incremental cycling test.

	T_1_	T_2_	T_3_	*P* value ANOVA	ICC	*p*	SEM	SWC	MDC
T_exh_ (min:s)	10:34 ± 3:02	11:19 ± 3:04	11:20 ± 3:22	0.5980	0.94	< .0001	0:51	1:08	1:41
W_max_ (W)	254.8 ± 56.2	264.3 ± 59.1	265.5 ± 64.1	0.7174	0.95	< .0001	14.5	21.8	28.8
HR_max_ (bpm)	172 ± 12	176 ± 8	176 ± 11	0.3477	0.49	< .0001	7.6	2.9	15.0
T_GET_ (min:s)	7:53 ± 2:09	8:19 ± 2:05	8:10 ± 2:09	0.5411	0.94	< .0001	0:35	0:46	1:08
W_GET_ (W)	207.1 ± 47.3	215.5 ± 43.1	210.7 ± 47.4	0.6710	0.93	< .0001	13.2	16.5	26.0
HR_GET_ (bpm)	163 ± 9	164 ± 10	162 ± 11	0.8140	0.79	< .0001	4.4	3.0	8.8
VO_2GET_ (mL/min)	2763 ± 675	2850 ± 594	2813 ± 657	0.7654	0.88	< .0001	234	227	462
VO_2peak_ (mL/min)	3212 ± 778	3256 ± 771	3300 ± 872	0.8594	0.96	< .0001	177	294	351
VCO_2peak_ (mL/min)	3522 ± 845	3684 ± 846	3739 ± 959	0.4010	0.92	< .0001	269	319	533
EE (kcal)	126 ± 64	138 ± 67	141 ± 72	0.6292	0.96	< .0001	14	25	28

EE, energy expenditure; HR_GET_, heart rate at gas exchange threshold; HR_max_, maximal heart rate; ICC, interclass correlation coefficient; MDC, minimal detectable change; SEM, standard error of measurement; SWC, smallest worthwhile change; T_exh_, time to exhaustion; T_GET_, time to gas exchange threshold; VCO_2peak_, maximal carbon dioxide production; VO_2peak_, peak oxygen uptake; VO_2GET_, oxygen uptake at gas exchange threshold; W_max_, maximal workload; W_GET_, workload at gas exchange threshold.

### FGB and ICT relationship

Correlations between FGB scores and ICT parameters were significant for all measurements besides HR_GET_ (Table 5). Strong correlations (> 0.7) were observed for FGB_R-1_ with T_exh_, W_max_, T_GET_, and EE; and for FGB_TOTAL_ with T_exh_ and EE. Moderate (0.5–0.7) correlations were found for FGB_R-1_ with W_GET_, VO_2GET_, VO_2peak_, and VCO_2peak_, for FGB_R-2_ with T_exh_, W_max_, T_GET_, W_GET_, VO_2peak_, VCO_2peak_, and EE, for FGB_R-3_ with T_exh_, W_max_, T_GET_, W_GET_, VO_2peak_, and EE, and for FGB_TOTAL_ with W_max_, T_GET_, W_GET_, VO_2GET_, VO_2peak_, and VCO_2peak_. In addition, correlations were significant but low for FGB_R-1_ with HR_max_, FGB_R-2_ with HR_max_, and VO_2GET_, FGB_R-3_ with HR_max_, HR_GET_, VO_2GET_, and VCO_2peak_, as well as FGB_TOTAL_ with HR_max_.

**Table 5:** Correlations between Fight Gone Bad performance and aerobic capacity.

	FGB_R-1_		FGB_R-2_		FGB_R-3_		FGB_TOTAL_	
	R^2^	*p*	R^2^	*p*	R^2^	*p*	R^2^	*P*
Incremental cycling test								
T_exh_ (min:s)	.7382	.000	.6981	.000	.6462	.000	.7175	.000
W_max_ (W)	.7197	.000	.6691	.000	.6108	.000	.6899	.000
HR_max_ (bpm)	.3568	.004	.3208	.010	.3221	.010	.3442	.006
T_GET_ (min:s)	.7051	.000	.6553	.000	.6049	.000	.6777	.000
W_GET_ (W)	.6795	.000	.6114	.000	.5619	.000	.6401	.000
HR_GET_ (bpm)	.2340	.065	.2065	.104	.2512	.047	.2364	.062
VO_2GET_ (mL)	.5771	.000	.4884	.000	.4310	.000	.5195	.000
VO_2peak_ (mL)	.6343	.000	.5538	.000	.5020	.000	.5851	.000
VCO_2peak_ (mL)	.6367	.000	.5533	.000	.4927	.000	.5831	.000
EE (kcal)	.7361	.000	.6909	.000	.6353	.000	.7110	.000

EE, energy expenditure; FGB_R-1_, Fight Gone Bad Round 1; FGB_R-2_, Fight Gone Bad Round 2; FGB_R-3_, Fight Gone Bad Round 3; FGB_TOTAL_, Fight Gone Bad total number of repetitions; HR_GET_, heart rate at gas exchange threshold; HR_max_, maximal heart rate; T_exh_, time to exhaustion; T_GET_, time to gas exchange threshold; VCO_2peak_, maximal carbon dioxide production; VO_2peak_, peak oxygen uptake; VO_2GET_, oxygen uptake at gas exchange threshold; W_max_, maximal workload; W_GET_, workload at gas exchange threshold.

**Figure 1:**
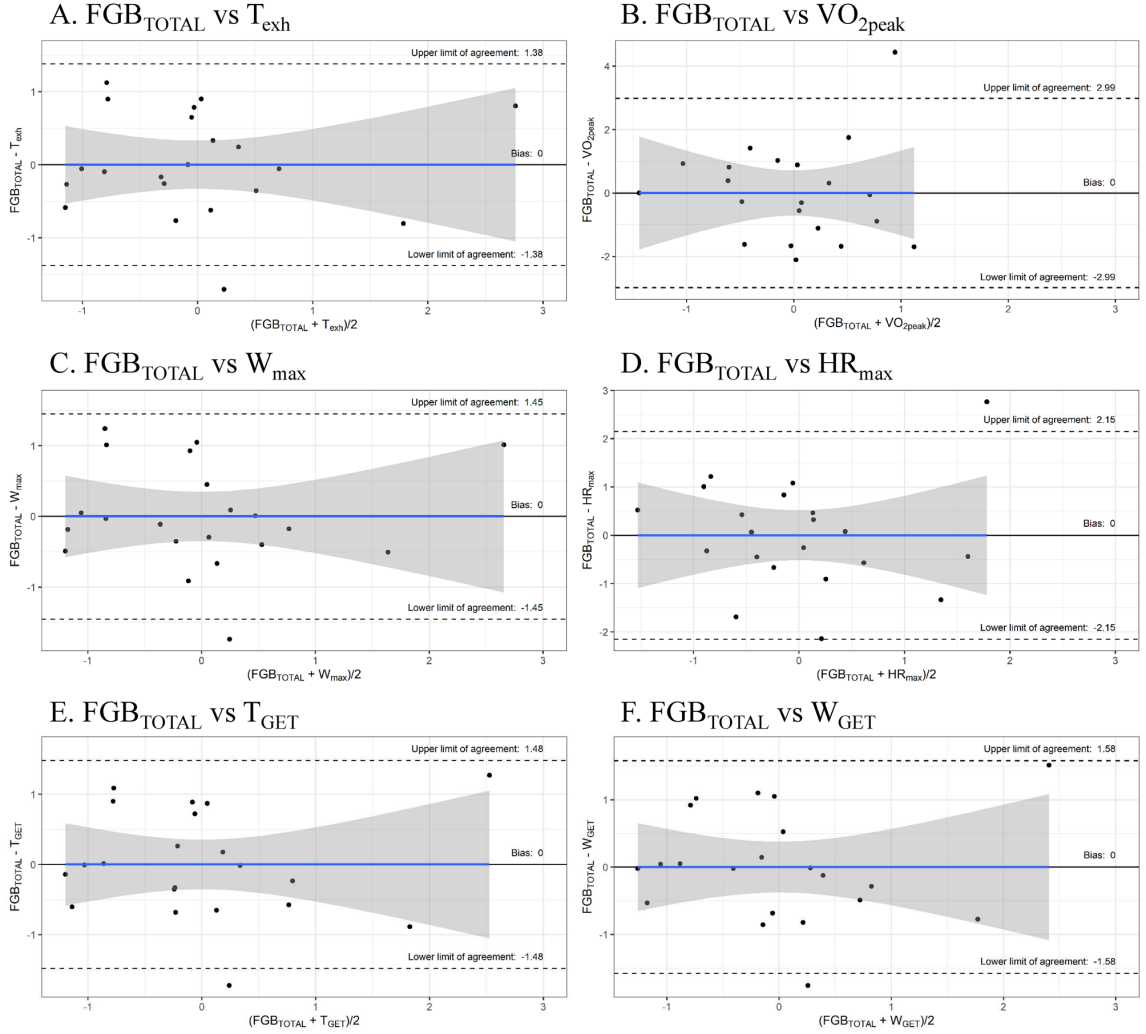
Bland–Altman plots for standardized measures of Fight Gone Bad performance and aerobic capacity. FGB_TOTAL_, Fight Gone Bad total number of repetitions; HR_max_, maximal heart rate; T_exh_, time to exhaustion; T_GET_, time to gas exchange threshold; W_max_, maximal workload; W_GET_, workload at gas exchange threshold; VO_2peak_, peak oxygen uptake.

The agreement of two methods was also assessed using the Bland–Altman method (Fig. 1). Bias for standardized FGB_TOTAL_ and performance in ICT measured as standardized T_exh_, VO_2peak_, W_max_, and HR_max_ were 0.0 ± 0.70, 0.0 ± 1.53, 0.0 ± 0.74 and 0.0 ± 0.10, respectively (Fig. 1A–D). Bias for standardized FGB_TOTAL_ and GET measured as standardized T_GET_ and W_GET_ were 0.0 ± 0.76 and 0.0 ± 0.81, respectively (Fig. 1E,F).

### Blood sample analysis

Biochemical marker analysis in blood revealed that MON count and HGB concentration were significantly different among three measurements in both FGB_PRE_ and FGB_POST_, whereas Pa concentrations were significantly different only in FGB_PRE_ (Table 6). ICC was rated as excellent in FGB_POST_ RBC, as good in FGB_PRE_ RBC, PLT, and HTC and in FGB_POST_ HTC and GLU, acceptable in FGB_PRE_ WBC, LYM, GRA and CK, and in FGB_POST_ WBC, LYM, GRA, PLT, La, Pa, CK, and LDH. ICC was significant but low in FGB_PRE_ HGB and LDH, and non-significant in FGB_PRE_ MON, GLU, La, Pa, and FGB_POST_ MON, and HGB. Apart from FGB_PRE_ WBC, HTC and FGB_POST_ RBC, for all blood measures SEM > SWC both FGB_PRE_ and FGB_POST_.

**Table 6:** Repeatability of hematological and biochemical parameters measured before and after Fight Gone Bad.

	T_1_	T_2_	T_3_	*P* value—ANOVA	ICC	*p*	SEM	SWC	MDC
**FGB**_**PRE**_									
WBC (10^9^/L)	8.42 ± 1.83	8.51 ± 1.85	7.92 ± 1.65	0.5697	0.63	< .0001	0.48	1.94	2.92
LYM (10^9^/L)	3.18 ± 0.94	3.46 ± 0.92	2.96 ± 0.55	0.2054	0.48	< .0001	0.54	0.21	1.07
MON (10^9^/L)	0.78 ± 0.28	1.04 ± 0.73	0.47 ± 0.16	0.0048	0.15	0.1020	0.47	0.11	0.94
GRA (10^9^/L)	4.46 ± 1.43	4.0 ± 1.61	4.50 ± 1.36	0.6040	0.52	< .0001	0.97	0.39	1.93
RBC (10^12^/L)	5.75 ± 0.24	5.84 ± 0.29	5.78 ± 0.28	0.5354	0.78	< .0001	0.10	0.07	0.20
HGB (mmol/L)	10.31 ± 0.26	10.53 ± 0.45	10.79 ± 0.26	0.0003	0.24	< .0001	0.35	0.09	0.69
PLT (10^9^/L)	293 ± 56	278 ± 62	281 ± 64	0.7036	0.73	< .0001	30.1	17.9	59.6
HTC (%)	40.78 ± 3.86	40.31 ± 4.38	39.78 ± 3.16	0.7118	0.74	< .0001	0.79	1.04	1.56
GLU (mg/dL)	116 ± 16	126 ± 30	109 ± 11	0.0605	0.15	0.1110	21.3	5.0	42.3
La (mmol/L)	2.28 ± 1.56	1.95 ± 0.65	1.62 ± 0.47	0.1076	0.00	0.4700	1.01	0.19	2.00
Pa (mmol/L)	0.43 ± 0.12	0.38 ± 0.10	0.35 ± 0.07	0.0254	-0.02	0.5400	0.11	0.02	0.22
CK (U/L)	313 ± 119	310 ± 79	312 ± 101	0.9554	0.45	< .0001	74.0	27.0	146.5
LDH (U/L)	368 ± 98	345 ± 86	382 ± 150	0.7251	0.34	< .0001	95.0	29.8	188.0
**FGB**_**POST**_									
WBC (10^9^/L)	12.41 ± 2.68	12.46 ± 2.15	12.13 ± 2.80	0.8811	0.57	< .0001	1.53	0.68	3.03
LYM (10^9^/L)	5.55 ± 1.33	6.03 ± 1.04	5.40 ± 1.25	0.2581	0.40	< .0001	0.91	0.31	1.79
MON (10^9^/L)	1.09 ± 0.37	1.46 ± 0.94	0.73 ± 0.23	0.0055	0.14	0.1130	0.61	0.14	1.21
GRA (10^9^/L)	5.76 ± 2.04	4.95 ± 2.05	6.0 ± 1.82	0.3244	0.55	< .0001	1.28	0.54	2.53
RBC (10^12^/L)	5.70 ± 0.24	5.73 ± 0.27	5.73 ± 0.27	0.9389	0.95	< .0001	0.05	0.07	0.09
HGB (mmol/L)	10.2 ± 0.27	10.3 ± 0.36	10.6 ± 0.26	0.0004	0.28	0.0159	0.31	0.09	0.62
PLT (10^9^/L)	333 ± 68	320 ± 66	325 ± 78	0.8188	0.61	< .0001	41.1	19.4	81.4
HTC (%)	41.69 ± 3.87	41.81 ± 3.97	41.43 ± 3.42	0.7118	0.82	< .0001	0.02	0.01	0.03
GLU (mg/dL)	159 ± 47	156 ± 48	161 ± 41	0.9581	0.83	< .0001	18.0	14.2	35.6
La (mmol/L)	13.19 ± 3.38	14.25 ± 3.10	13.84 ± 4.59	0.6372	0.60	< .0001	2.32	1.08	4.59
Pa (mmol/L)	0.80 ± 0.14	0.84 ± 0.16	0.77 ± 0.15	0.5136	0.41	< .0001	0.12	0.04	0.23
CK (U/L)	330 ± 120	336 ± 103	348 ± 119	0.9667	0.52	< .0001	80.3	32.5	159.1
LDH (U/L)	378 ± 83	394 ± 116	411 ± 96	0.6949	0.58	< .0001	61.8	27.5	122.3

CK, creatine kinase; FGB_POST_, after Fight Gone Bad; FGB_PRE_, before Fight Gone Bad; GLU, glucose; GRA, granulocytes; HGB, hemoglobin; HTC, hematocrit; ICC, interclass correlation coefficient; La, lactate; LDH, lactate dehydrogenase; LYM, lymphocytes; MON, monocytes; MDC, minimal detectable change; Pa, pyruvate; PLT, platelets; RBC, red blood cells; SEM, standard error of measurement; SWC, smallest worthwhile change; WBC, white blood cells.

Blood parameters ICT_PRE_ and ICT_POST_ were also measured. HGB concentrations were significantly different among three measurements in both ICT_PRE_ and ICT_POST_, whereas GLU concentrations were significantly different only ICT_PRE_ (Table 7). ICC was excellent for RBC ICT_PRE_ and ICT_POST_, good in ICT_PRE_ PLT and ICT_POST_ PLT, and HTC, acceptable in ICT_PRE_ WBC, LYM, GRA, HTC, La, and LDH, and in ICT_POST_ WBC, LYM, MON, GRA, GLU, and La. Low but significant ICC was observed in ICT_PRE_ MON, HGB and CK, and in ICT_POST_ HGB, Pa, CK, and LDH. Non-significant ICC were found in ICT_PRE_ GLU, and Pa. SEM < SWC was observed only in pre- and ICT_POST_ RBC.

**Table 7:** Repeatability of hematological and biochemical parameters measured before and after incremental cycling test.

	T_1_	T_2_	T_3_	*P* value—ANOVA	ICC	*p*	SEM	SWC	MDC
**ICT**_**PRE**_									
WBC (10^9^/L)	7.33 ± 1.79	7.44 ± 1.89	8.35 ± 2.93	0.2269	0.57	< .0001	1.47	0.64	2.92
LYM (10^9^/L)	2.61 ± 0.62	2.63 ± 0.62	2.69 ± 0.74	0.8891	0.54	< .0001	0.45	0.19	0.89
MON (10^9^/L)	0.61 ± 0.24	0.54 ± 0.16	0.51 ± 0.24	0.3447	0.36	0.0031	0.18	0.06	0.35
GRA (10^9^/L)	4.11 ± 1.39	4.28 ± 1.57	5.14 ± 2.76	0.1468	0.48	< .0001	1.39	0.53	2.75
RBC (10^12^/L)	5.80 ± 0.27	5.83 ± 0.28	5.83 ± 0.28	0.9004	0.96	< .0001	0.05	0.10	0.11
HGB (mmol/L)	10.57 ± 0.22	10.61 ± 0.19	10.86 ± 0.28	0.0004	0.23	0.0219	0.23	0.06	0.46
PLT (10^9^/L)	269 ± 54	275 ± 57	271 ± 58	0.9654	0.85	< .0001	22.0	18.6	43.6
HTC (%)	40.37 ± 2.87	40.21 ± 3.74	40.23 ± 2.21	0.9728	0.69	< .0001	0.02	0.01	0.03
GLU (mg/dL)	105 ± 15	110 ± 18	118 ± 20	0.0495	0.08	0.2330	17.5	3.75	34.7
La (mmol/L)	1.46 ± 0.52	1.56 ± 0.66	1.75 ± 0.80	0.4952	0.43	0.0005	0.52	0.19	1.03
Pa (mmol/L)	0.35 ± 0.09	0.34 ± 0.08	0.36 ± 0.12	0.8226	0.03	0.4010	0.10	0.02	0.20
CK (U/L)	396 ± 168	311 ± 96	327 ± 147	0.1179	0.33	0.0040	115.4	35.4	228.5
LDH (U/L)	379 ± 148	358 ± 92	346 ± 97	0.7357	0.46	0.0003	85.8	31.8	170.0
**ICT**_**POST**_									
WBC (10^9^/L)	10.54 ± 2.62	11.80 ± 3.14	12.01 ± 3.39	0.2030	0.59	< .0001	1.91	0.87	3.78
LYM (10^9^/L)	4.55 ± 1.51	5.01 ± 1.45	4.80 ± 1.20	0.6044	0.47	0.0002	0.96	0.36	1.90
MON (10^9^/L)	0.80 ± 0.29	0.87 ± 0.35	0.81 ± 0.34	0.8105	0.44	0.0005	0.24	0.09	0.48
GRA (10^9^/L)	5.21 ± 1.53	5.92 ± 229	6.41 ± 3.3	0.2098	0.46	0.0002	1.73	0.63	3.42
RBC (10^12^/L)	5.77 ± 0.27	5.74 ± 0.27	5.75 ± 0.27	0.9815	0.97	< .0001	0.05	0.09	0.09
HGB (mmol/L)	10.43 ± 0.26	10.34 ± 0.23	10.68 ± 0.26	0.0035	0.32	0.0051	0.23	0.07	0.45
PLT (10^9^/L)	288 ± 59	303 ± 61	290 ± 69	0.7770	0.79	< .0001	29.7	20.7	58.8
HTC (%)	42.81 ± 3.43	43.05 ± 3.75	43.12 ± 3.45	0.9606	0.88	< .0001	0.01	0.01	0.02
GLU (mg/dL)	132 ± 36	131 ± 28	127 ± 24	0.8492	0.66	< .0001	17.9	9.17	35.4
La (mmol/L)	11.69 ± 3.57	13.52 ± 2.83	13.35 ± 2.06	0.1097	0.56	< .0001	1.94	0.84	3.84
Pa (mmol/L)	0.74 ± 0.13	0.78 ± 0.12	0.79 ± 0.10	0.2688	0.30	0.0112	0.10	0.03	0.20
CK (U/L)	399 ± 189	327 ± 104	362 ± 149	0.2608	0.37	0.0022	120.9	39.2	239.4
LDH (U/L)	365 ± 97	404 ± 112	414 ± 82	0.2822	0.33	0.0049	80.7	25.0	159.7

CK, creatine kinase; GLU, glucose; GRA, granulocytes; HGB, hemoglobin; HTC, hematocrit; ICC, interclass correlation coefficient; ICT_POST_, after incremental cycling test; ICT_PRE_, before incremental cycling test; La, lactate; LDH, lactate dehydrogenase; LYM, lymphocytes; MON, monocytes; MDC, minimal detectable change; Pa, pyruvate; PLT, platelets; RBC, red blood cells; SEM, standard error of measurement; SWC, smallest worthwhile change; WBC, white blood cells.

In the present study we also evaluated the differences in means (T_1_, T_2_, and T_3_) of blood parameters between FGB_PRE_, FGB_POST_, ICT_PRE_, and ICT_POST_. We found that WBC, LYM, MON, La, and Pa were significantly higher FGB_POST_ than FGB_PRE_ and ICT_POST_ than ICT_PRE_ (Table 8). GRA were higher ICT_POST_ than ICT_PRE_. GLU was higher FGB_POST_ than FGB_PRE_. LYM were significantly different between FGB_PRE_ vs ICT_PRE_ and between ICT_POST_ and FGB_POST_. No significant differences were observed for RBC, HGB, PLT, CK and LDH.

**Table 8:** Differences in hematological and biochemical parameters between Fight Gone Bad and incremental cycling test (means of three measurements T_1_–T_3_).

	Mean FGB_PRE_	Mean FGB_POST_	Mean ICT_PRE_	Mean ICT_POST_	*P*-value ANOVA FGB_PRE_-versus FGB_POST_	*P*-value ANOVA ICT_PRE_ versus ICT_POST_	*P*-value ANOVA FGB_PRE_ versus ICT_PRE_	*P*-value ANOVA FGB_POST_ versus ICT_POST_
WBC (10^9^/L)	8.29 ± 1.81	12.30 ± 2.48	7.80 ± 2.24	11.57 ± 2.98	< .0001	< .0001	1.0000	1.0000
LYM (10^9^/L)	3.20 ± 0.86	5.66 ± 1.21	2.67 ± 0.66	4.82 ± 1.32	< .0001	< .0001	0.0178	0.0139
MON (10^9^/L)	0.75 ± 0.53	1.09 ± 0.68	0.56 ± 0.22	0.83 ± 0.33	0.0072	0.0023	0.0849	0.2056
GRA (10^9^/L)	4.33 ± 1.50	5.53 ± 2.01	4.57 ± 1.92	5.92 ± 2.34	0.0703	0.0380	1.0000	1.0000
RBC (10^12^/L)	5.79 ± 0.27	5.72 ± 0.26	5.82 ± 0.28	5.75 ± 0.27	ns	ns	ns	ns
HGB (mmol/L)	10.55 ± 0.38	10.39 ± 0.35	10.68 ± 0.26	10.51 ± 0.28	ns	ns	ns	ns
PLT (10^9^/L)	282.0 ± 61.1	322.8 ± 70.8	270.0 ± 57.1	291.6 ± 65.2	ns	ns	ns	ns
GLU (mg/dL)	117.2 ± 22.3	153.0 ± 44.6	111.7 ± 18.3	128.3 ± 30.5	0.0123	0.3068	1.0000	0.1543
La (mmol/L)	1.92 ± 1.04	13.41 ± 3.52	1.53 ± 0.69	12.74 ± 2.93	< .0001	< .0001	0.0605	1.0000
Pa (mmol/L)	0.38 ± 0.11	0.79 ± 0.15	0.35 ± 0.10	0.77 ± 0.12	< .0001	< .0001	0.2477	1.0000
CK (U/L)	307.5 ± 100.5	331.1 ± 112.9	347.7 ± 141.2	367.1 ± 152.1	ns	ns	ns	ns
LDH (U/L)	356.7 ± 115.3	385.4 ± 100.3	356.5 ± 116.8	391.5 ± 98.8	ns	ns	ns	ns

CK, creatine kinase; FGB_POST_, after Fight Gone Bad; FGB_PRE_, before Fight Gone Bad; GLU, glucose; GRA, granulocytes; HGB, hemoglobin; ICT_POST_, after incremental cycling test; ICT_PRE_, before incremental cycling test; La, lactate; LDH, lactate dehydrogenase; LYM, lymphocytes; MON, monocytes; Pa, pyruvate; PLT, platelets; RBC, red blood cells; WBC, white blood cells.

## Discussion

Cross-training is still becoming more and more popular. A whole range of athletes, as well as sedentary people, can benefit from cross-training, because it gives multiple stimulus to the muscles, and all exercise can be scaled to meet an individual’s abilities and needs. Thus, there is a need to find a validated test to measure its performance. In this paper, we proposed a benchmark workout, Fight Gone Bad, to be such a test. FGB incorporates several of the physiological traits that are most crucial for HIFT performance, i.e., stamina, speed, strength, endurance, and power. The main findings showed that FGB gives reliable and repeatable results when performed three times with each measurement separated by 10 days from the others. Moreover, we revealed that FGB results were strongly correlated to aerobic fitness. When the results were standardized, we also found that the agreement of FGB with aerobic performance indices such as T_exh_, VO_2peak_, W_max_, HR_max_, T_GET_, and W_GET_ was high.

In practical and scientific respects, the reproducibility of a test is essential to determine whether an individual has experienced a training response. Moreover, reliability estimates the extent to which the change in the measured score is due to a change in the true score^14^. The present study is the first to investigate whether FGB performance is reproducible across repeated measurements. There were no differences in body composition, FGB, HR_FGB,_ and ICT between T_1_, T_2_, and T_3_. Relative reliability was measured as the interclass correlation coefficient. The ICC reflects a test's ability to differentiate between participants and, hence, the position of the individual relative to others in the group^15^. Relative reliability was found to be excellent for FGB_R-1_ and FGB_TOTAL_ and good for FGB_R-2_ and FGB_R-3_, showing the linearity of the relationship between the repeated measures. However, the ICC does not provide information about the accuracy of the scores for an individual. Therefore, absolute reliability was calculated as SEM. Lower SEM means the method is more precise^16^. SEM for FGB_R-1_ and FGB_TOTAL_ was 6% each, for FGB_R-2_—8% and for FGB_R-3_—9%. The smallest worthwhile changes (SWC) were higher than SEM for FGB_R-1_ and FGB_TOTAL_, indicating the ability of test to detect small and meaningful performance changes. Interestingly, the repeatability of body composition measurements in our study indicates that the participant did not implement any changes in their lifestyles throughout the study (i.e., body mass reduction), that could influence the performance.

Moreover, relative reliability was even better for ICT, where for most of the measured parameters (T_exh_, W_max_, T_GET_, W_GET_, VO_2peak_, VCO_2peak_ and EE), ICC was close to 1.0 and SEM was low. This is in accordance with previous studies. Dideriksen and Mikkelsen^17^ showed excellent ICC (< 0.9) for VO_2max_, W_max_, and HR_max_ and good ICC (0.7–0.9) for VO_2VT_ in recreationally trained triathletes (n = 13). Weston and Gabbett^18^ found ICC > 0.9 for VO_2max_, VE, VCO_2_, HR, and W, with measurement errors below 5% in trained cyclists (n = 16). Graded exercise testing is a reliable tool that is widely used for the determination of VO_2peak_ in sports performance, research, and clinical diagnostics^19^. However, this is beyond the scope of the present paper.

Considering speed and strength efforts, Fight Gone Bad (FGB) is a high-intensity workout of moderate duration (17 min in total). It is performed very fast and demands a high level of muscular endurance. The present study compared the results in FGB to aerobic fitness measured in incremental cycling test. We found strong and moderate correlations between FGB performance and time to exhaustion, maximum workload, VO_2peak_, VCO_2peak_, time to GET, and workload at GET. This suggests that aerobic fitness is crucial to FGB performance. This might seem counterintuitive, since the HR observed during each round of FGB were very close to HR_max_ measured in ICT, showing that the effort put forth by the participants in FGB was extremely high. What is more, given that HR at gas exchange threshold was at the level of 162 ± 11 bpm to 164 ± 10 bpm, we can assume that work done in FGB rounds was mainly of an anaerobic nature (HR mean of 3 rounds was between 169 ± 11 bpm to 173 ± 10 bpm). One explanation, though not strong enough, for such a phenomenon can be that the duration of FGB forces the engagement of the aerobic energy system. The other reason for that can be in the main characteristic of FGB, which is the 1-min rest periods between the rounds. It seems that the ability to recover between bouts of exercise is dependent upon oxidative capacity^20^. One study showed that better recovery between repeated bouts of Wingate sprints was associated with better cross-training performance^20^. Therefore, it suggests that even if rounds in FGB are to some extent anaerobic in nature, the ability to sustain effort throughout the entire FGB may be reliant on the aerobic recovery efficiency during breaks between the rounds. This is in accordance with literature suggesting that aerobic capacity enhances recovery from high intensity intermittent exercise through increased aerobic response, improved lactate removal, and enhanced phosphocreatine regeneration^21^.

Some investigators aimed to compare different physiological variables with HIFT performance. Bellar et al.^8^ found that cross-training performance was correlated to aerobic power (VO_2max_) but only in experienced athletes (r = 0.453, *p* = 0.03) and not in the naïve (r = 0.168, *p* = 0.64). The cross-training workout they used consisted of 21–15-9 repetitions of (1) sumo deadlift high pull, (2) box jump (50 cm), and (3) 40-m farmer's walk gripping two 20-kg bumper plates. The score was the time for workout completion. In yet another paper, Dexheimer et al.^10^ showed that the higher the VO_2max_ the better the result in ‘Fran’, ‘Nancy’, and ‘CrossFit Total’. However, in the regression model, VO_2max_ explained 68% of the variance only in ‘Nancy’^10^. ‘Fran’ consisted of 3 rounds of 21–15-9 repetitions of thrusters and pull-ups. ‘Nancy’ was a workout of 5 rounds of a 400 m run and 15 overhead squats with a barbell (95 lb men/65 lb women). ‘CrossFit Total’ included 1-repetition maximum (RM) back squat, strict shoulder-press, and deadlift. Interestingly, they did not find any significant association between VO_2max_ and the ‘Grace’ workout (30 clean and jerks (135 lb men/95 lb women)). The authors thus hypothesized that those with higher VO_2max_ may perform better in longer workouts that require running (i.e., ‘Nancy’) compared to shorter (i.e., ‘Grace’). In contrast to Dexhaimer, Butcher et al.^9^ found no correlation between VO_2max_ and ‘Fran’, ‘Grace’, or ‘Cindy’ (as many rounds as possible of 5 repetitions of pull-ups, 10 repetitions of push-ups, and 15 repetitions of bodyweight squats performed in 20 min) or ‘CrossFit Total’. However, they found significant correlations between VO_2_ at anaerobic threshold and ‘Fran’, ‘Grace’, and ‘CrossFit Total’. Furthermore, Martinez-Gomez et al.^11^ aimed to determine which physiological variables could predict performance during a cross-training competition (The Open, 2019). They found that the combination of lower-body muscle power (squat jump performance), reactive strength (reactive strength index during a drop jump), and aerobic power (as measured with the VO_2max_) together explained 81% of the cross-training performance variance, showing that HIFT performance is associated with a variety of fitness markers related to both aerobic and anaerobic/power capabilities. This seems to confirm that HIFT demands high adaptation to both aerobic and anaerobic types of exercise. Therefore, the improvement in cardiorespiratory fitness may enhance the performance in longer workouts like FGB or ‘Nancy’. Thus, it would be reasonable to include aerobic training in cross-training programming. Moreover, it would be also beneficial to include training like FGB in sports using varying energy systems like combat and team sports. In these sports, all three energy systems are used according to the intensity, rhythm, and duration of the competition. That is why they can benefit the most from FGB, which is of high intensity yet strongly correlated with aerobic fitness (i.e., T_exh_).

Also, the present study is the first to provide extensive data on biochemical response after each exercise test among HIFT-trained participants. La and Pa concentrations significantly increased FGB_POST_ and ICT_POST_ compared to FGB_PRE_ and ICT_PRE_, respectively, but there were no differences in La or Pa between FGB_POST_ (13.41 ± 3.52 mmol/L and 0.79 ± 0.15 mmol/L, respectively) and ICT_POST_ (12.74 ± 2.93 mmol/L and 0.77 ± 0.12 mmol/L, respectively). GLU concentration increased significantly only FGB_POST_ versus FGB_PRE_ (153.0 ± 44.6 mg/dL to 117.2 ± 22.3 mg/dL). In a previous study, Feito et al.^20^ observed similar concentrations of La in the last round of 15-min cross-training AMRAP (13.89 ± 2.23 mmol/L in males and 11.53 ± 1.69 mmol/L in females). The 15-min AMRAP circuit consisted of 250-m of rowing, 20 Kettle bell swings (16 kg for men and 12 kg for women), and 15 dumbbell thrusters (16 kg for men and 9 kg for women). In another study, La and GLU concentrations were significantly different between WOD_1_ and WOD_2_ (13.30 ± 1.87 mmol/L La after WOD_1_ vs 18.38 ± 2.02 mmol/L La after WOD_2_, 135.4 ± 19.6 mg/dL GLU after WOD_1_ vs 167.4 ± 19.6 mg/dL GLU after WOD_2_)^22^. WOD_1_ (AMRAP) consisted of as many rounds as possible of burpees and toes to bar increasing repetitions (1–1, 2–2, 3–3…) in five minutes. WOD_2_ was the number rounds for time (RFT) consisting of three rounds of 20 repetitions of wall ball (9 kg) and 20 repetitions of power clean (a load of 40% 1RM) in the shortest possible time. HR measurement showed that during WOD_1_ the majority of time was spent in the intensity zone of 50–59% HR_max_, whereas during WOD_2_ it was in the zone of 90–100% HR_max_. Thus, this explains the difference in La and GLU concentration. Fernandez-Fernandez et al.^23^ observed that La concentration increased from 4.0 ± 1.3 mmol/L to 14.5 ± 3.2 mmol/L in ‘Cindy’ and from 4.0 ± 1.3 mmol/L to 14.0 ± 3.3 mmol/L in ‘Fran’. Moreover, Tibana et al.^24^ observed lower concentrations of La after two different cross-training workouts, 11.84 ± 1.34 mmol/L after WOD_1_ and 9.05 ± 2.56 mmol/L after WOD_2_. WOD_1_ was 10 min of AMRAP of 30 double-unders and 15 power snatches (34 kg)^24^. WOD_2_ was 12 min AMRAP of rowing 250 m and 25 target burpees. They also observed an increase in GLU concentration (81.59 ± 10.27 mg/dL to 114.99 ± 12.52 mg/dL in WOD_1_ vs. 69.47 ± 6.97 mg/dL to 89.95 ± 19.26 mg/dL in WOD_2_).

In the present study, we did not observe any significant differences in CK and LDH activities. Timon et al.^22^ noted a significant increase in CK activity after two WODs (from 566.4 ± 159.1 IU/L to 689.6 ± 281.9 IU/L in WOD_1_ (5 min AMRAP) and from 406.8 ± 201.0 IU/L to 492.2 ± 203.8 IU/L in WOD_2_ (RFT)). CK remained elevated 24 h post exercise (864.0 ± 369.5 IU/L after WOD_1_ and 673.8 ± 444.1 IU/L after WOD_2_) and returned to baseline 48 h post exercise. However, they did not note any differences in LDH.

Finally, it has been generally shown that acute exercise increases erythrocytes, leucocytes and platelet counts, hematocrit values, and hemoglobin concentration significantly as compared to pre-exercise values, and these increments depend on fluid shifts (plasma volume contraction) caused by the exercise^25^. For this reason, we standardized post exercise biochemical and hematological parameters in relation to the hematocrit value. We observed significant differences in HGB and MON between T_1_, T_2_ and T_3,_ yet they were not clinically important. WBC, LYM, and MON concentration increased after exercise (both FGB and ICT). GRA increased significantly only after ICT. Interestingly, LYM count was higher FGB_POST_ than ICT_POST_, but pre-exercise values were also higher before FGB than before ICT. The increase in WBC can be attributed to increased blood flow that recruits the leukocytes from the marginal pool and/or hormonal changes which are likely to be mediated by beta-2 adrenergic receptors^25^. In contrast to our study, in sedentary adults acute high-intensity interval training (HIIT) increased HGB concentration from 15.75 ± 0.76 g/dL pre-exercise to 16.59 ± 0.81 g/dL post-exercise and RBC count from 5.44 ± 0.22 × 10^12^/L to 5.92 ± 0.22 × 10^12^/L^26^. In addition, they observed a significant increase in WBC (from 7.32 ± 1.83 × 10^9^/L to 12.84 ± 3.37 × 10^9^/L) and LYM (3.11 ± 1.59 × 10^9^/L to 5.22 ± 1.99 × 10^9^/L) but not in MON. The differences between individual studies most often result from not using the hematocrit conversion formula that takes into account post-exercise dehydration and the transfer of fluids from the bloodstream to the tissues^27^. This can explain the differences in blood cell counts observed by Belviranli et al.^26^.

Our study is the first to evaluate the repeatability of a cross-training test. The strength of our study is that the participants performed FGB and ICT three times in the same conditions, which allowed more precise assessment of reproducibility. Moreover, we controlled whether the participants implemented any significant changes in their lifestyles, i.e., reduction diet, by measuring body composition. We also controlled the HR during the test to evaluate the intensity of exercise. By comparing average FGB HR to HR_max_ measured in ICT, we could assume that FGB was very intense and that every time the participants put in a lot of effort. We also controlled the biochemical and hematological parameters throughout the study, which gave us insight into metabolic changes in relation to both exercise tests.

The biggest limitation of our study is that we only measured the correlation of FGB with aerobic fitness. HIFT is varied in its nature, because it combines strength, power, speed, agility, and cardiovascular fitness. For this reason, we claim that future studies should evaluate the relationship between FGB performance and other physiological parameters such as anaerobic power or strength. Even though our study shows that FGB gives reliable scores, it seems important to evaluate its connection with other physical traits. It should also be taken into account that we used a cycloergometer for aerobic fitness evaluation in this study, which, to some extent, could affect the obtained aerobic fitness results. It is well known that aerobic power measured on a treadmill is higher than on a cycloergometer, because running engages whole-body and cycling mostly lower-body movements. However, the choice of a cycloergometer was motivated by the reluctance of our study group towards running. Thus, there was a justified concern that the participants would not fully engage in a graded running test. It is also worth-noting that the participants were well adapted to perform lower-body movements (like squats, deadlifts and lunges), which could be beneficial for cycling performance.

## Conclusions

Our study showed that Fight Gone Bad is a reliable and repeatable test to measure cross-training performance. Moreover, FGB is strongly correlated with aerobic fitness. FGB can be used as a tool in interventional studies to evaluate the changes in cross-training scores. Furthermore, given that FGB is a non-invasive, easy to perform, and accessible test, it can be regularly used by coaches throughout the training season.

## Methods

### Participants

**Figure 2:**
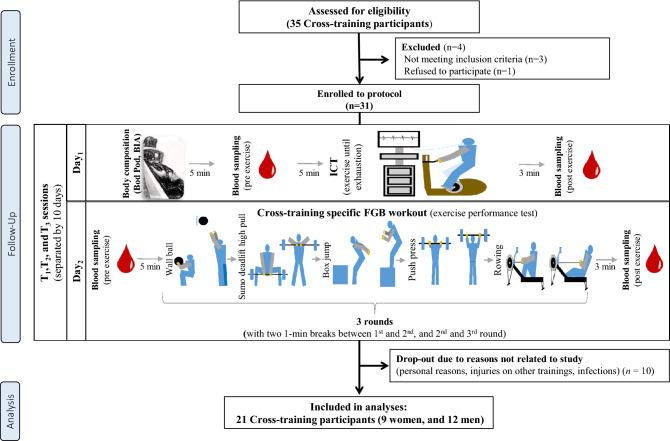
A flow chart of the study design.

Thirty-one participants were initially enrolled in this study. However, twenty-one (9 women, and 12 men with mean ± SD ages of 31.5 ± 5.5 years, body height 174 ± 8 cm, baseline values of body mass 73.0 ± 14.0 kg, free fat mass (FFM) 58.7 ± 13.9 kg, fat mass (FM) 19.1 ± 7.1%, and VO_2max_ 3191 ± 823 mL/min) completed the entire study protocol and were included in the analyses (Fig. 2). The participants were at a similar moderate athletic level. They have been regularly doing HIFT at Rankor Athletics, Reebok CrossFit Poznań, and Caffeine Barbell clubs in Poznań, Poland. The criteria to qualify for the study included the following: age between 20 and 40 years, the absence of injury and/or any other issues, good health with a valid and up-to-date medical certificate confirming the athlete’s ability to practice sports, at least 2 years of regular cross-training experience, and a minimum of 4 workout sessions (cross-training) per week for at least six months. We included both males and females in order to have equal participation of both genders in HIFT training and to test gender-related impact, assuming the purpose and scope of this work was considered negligible. Exclusion criteria included the following: being a current smoker, participating in illicit drug use, alcohol consumption greater than the equivalent 1–2 one alcoholic drinks per week, and dietary supplement use or being on any special diet within 3 weeks of the study’s commencement. For females, additional exclusion criteria were being pregnant or planning to become pregnant during the study. The cross-training box coaches enabled confirmation of the required inclusion criteria declared by the participants. They also supported the control of training adherence compliance. The drop-outs were predominantly independent from the study protocol (Fig. 2). The reasons for dropouts were as follows: personal, infections, minor injuries during customary training, and/or the inability to participate in the time frame of the planned protocol. The studies were conducted in 2015 and 2016 off season. All subjects declared that they had not introduced any changes in their lifestyles, elements of training, and/or customary nutrition.

The study protocol was reviewed and approved by the local ethical committee (Bioethics Committee at Poznan University of Medical Sciences, Poznan, Poland). Each subject was informed of the testing procedure, its purpose, and the risks of the study. Each participant submitted her/his written consent to participate. All procedures were conducted in accordance with the ethical standards of the 1975 Helsinki Declaration.

### Study design and protocol

The primary outcomes in this paper were the repeatability and reliability of FGB performance and its relation to aerobic performance. The study protocol included three visits to the Exercise Tests Laboratory at the of the Department of Human Nutrition and Dietetics (DHND) at the Poznan University of Life Sciences and selected “Cross-training Boxes” in Poznan at baseline (T_1_), and after 10 (T_2_) and 20 (T_3_) days, respectively (Fig. 2). Subjects were instructed not to participate in any high-intensity or long-duration training sessions at least 24 h before testing. All measurements at the DHND were performed in the morning (7.30–10.00 AM) and in a fasting state (water intake was recommended; a standardized meal was eaten the previous night immediately before going to sleep (about 1.2 g of carbohydrates per kg of body mass and 40 g of protein)). At the beginning, subjects underwent body composition analysis. Afterward, an incremental cycling test until volitional exhaustion was performed. During all of these measurements, the ambient temperature remained at 20‒22 °C. In the afternoon of the next day and three hours after standardized small meals (about 0.6 g of carbohydrates per kg of body mass and 15 g of protein), the discipline-specific cross-training test was performed. Enrolled participants were familiar with the tests and procedures used as they had participated in some previous research projects.

### Anthropometry and body composition

Body mass (kg) and height (cm) were measured using a professional medical scale with a stadiometer (WPT 60/150 OW, RADWAG, Radom, Poland) at an accuracy of 0.1 cm and 0.1 kg for height and body mass, respectively. FM and FFM were assessed based on air displacement plethysmography using the Bod Pod (Cosmed, Rome, Italy) as described previously^12,13^. Total body water and hydration level and additional FM and FFM evaluation was assessed by bioelectric impedance with Bodystat 1500 (Bodystat Inc, Douglas, UK) based on the previously mentioned recommended procedures^28^.

### Exercise tests

The study protocol consisted of the incremental cycling test (ICT) and FGB workout performed 3 times (T_1_, T_2_, and T_3_). Between ICT and FGB tests at least a 30-h recovery break was implemented. Prior to each tests (ICT and FGB), participants were given instructions on the procedure, and they completed a brief warm-up period (a 5-min effort on a cycloergometer (Kettler-X1, Kettler, Ense-Parsit, Germany) of approximately 50 W power and ~ 70 rpm cadence, followed by a 5-min light stretching and 5-min break). All tests were performed in proper workout clothing and shoes, and the tests were supervised by an experienced researcher. Heart rate was continuously monitored during exercise using a telemetric system (Polar, Kempele, Finland). Furthermore, capillary blood samples were obtained for analysis before and after each test. During exercise, all test participants were verbally encouraged to maximize their efforts.

### Aerobic fitness test

An exercise test on the Kettler X1 cycloergometer (Kettler, Ense-Parsit, Germany) was performed to determine peak oxygen uptake (VO_2peak_), and gas exchange threshold (GET). We considered the VO_2_peak to be the moment when the individual oxygen uptake (VO_2_) recorded during the ICT reached the highest point^29^. To determine the GET during the ICT, the V-slope method was applied based on an analysis of the linear regression for the curve of increasing CO_2_ exhalation in comparison to the curve of increasing O_2_ uptake^30–32^.The initial load was set at 50 W for women and 75 W for men and increased every 1.5 min by 25 W until volitional exhaustion. Respiratory parameters and heart rate (HR) were measured (breath by breath) by the Quark CPET ergospirometer (Cosmed, Rome, Italy). Measured variables included time to exhaustion (T_exh_), maximal workload (W_max_), maximum heart rate (HR_max_), time to GET (T_GET_), workload at GET (W_GET_), heart rate at GET (HR_GET_), oxygen uptake at GET (VO_2GET_), VO_2peak_, peak carbon dioxide production (VCO_2peak_), and energy expenditure (EE).

### Fight Gone Bad

Fight Gone Bad comprised three rounds of five exercises: wall ball, sumo deadlift high pull, box jump, push press, and rowing^13,33,34^. Participants were instructed to complete as many repetitions as possible in one minute at each station prior to moving to the next station. After completing each of the five stations, participants had one minute of rest (Rest_1_ between the 1st and 2nd and Rest_2_ between the 2nd and 3rd rounds) before beginning the next round^13,34^. Wall balls combined a front squat with a medicine ball (6 kg for females, 9 kg for males) and a push press-like throwing of the ball to a target located 2.75 m for females and 3.0 m for males. At the bottom of the squat, the hips should be lower than the knees. In sumo deadlift high pull, the feet were wider than the hips, and the grip was inside the knees. The exercise started with lifting the bar (25 kg for females, 35 kg for males) from the ground like in classical deadlift, but then the bar was pulled to the chest. At the end, the elbows should be higher than the shoulders. The Box jump started with both feet on the ground. Athletes jumped on a box that was 50 cm tall for females and 65 cm for males with landing on both feet. The exercise ended when shoulders, hips, and knees were extended in one line. Push press started with lifting the bar (25 kg for females and 35 kg for males) from the ground to the front rack. Then the bar was pushed overhead using leg power. After the shoulders were straight, the bar was dropped back to the shoulders. Rowing was performed on an ergometer. Feet were taped to the feet plates with special straps. The handle was pulled towards the chest, using the push from the knees. The test was video recorded in order to allow an accurate count of all properly done repetitions. For each valid repetition, a participant needed to complete a full range of motion required for a specific exercise.

### Blood samples analysis

Blood was collected by qualified medical personnel in accordance with applicable procedures. Before (ICT_PRE_ and FGB_PRE_) and 3 min after exercise tests (ICT_POST_ and FGB_POST_) capillary blood was collected from a fingertip of the nondominant hand using a disposable lancet-spike Medlance Red (HTL-STREFA, Łódź, Poland) with a 1.5 mm blade and 2.0 mm penetration depth as described previously^13^. Approximately 300 μL of blood was collected into a Microvette CB 300 tube (Sarstedt, Nümbrect, Germany) containing K2-EDTA (EDTA dipotassium salt) as anticoagulant for hematological measurements. Blood sample tests were carried out with the use of an 18-parametric automated hematology analyzer Mythic 18 (Orphée, Geneva, Switzerland). The count of white blood cells (WBC), lymphocytes (LYM), monocytes (MON), granulocyte (GRA), red blood cells (RBC), platelets (PLT), as well hemoglobin (HGB) concentration and hematocrit (HTC) value were considered in the study. Furthermore, another 300 μL of capillary blood was collected in a Microvette CB 300 Z tube (Sarstedt, Nümbrect, Germany) with a clotting activator, in which the activities of creatine kinase (CK; EC 2.7.3.2; Liquick Cor-CK, Cormay, Cat No. 1-219, Łomianki Poland) and lactate dehydrogenase (LDH; EC 1.1.1.27; Liquick Cor-LDH, Cormay, Cat No. 1-239, Łomianki Poland) were measured using an optimized kinetic methods. In addition, 50 μl of capillary blood was collected into a neutral (without anticoagulant) glass capillary (Vitrex, Medlab, Raszyn, Poland). The blood samples were deproteinized in 0.6 mol/L of perchloric acid (HClO_4_). After centrifuging (4000 g/10 min/4 °C), the supernatant was isolated. The enzymatic measurements of lactate (La) and pyruvate (Pa) concentrations were based on methodology proposed by Maughan^35^. Glucose concentration was detected with an enzymatic, colorimetric PZ Cormay test (Liquick Cor-GLUCOSE, Cat No. 2-201, Łomianki Poland). All biochemical measurements were conducted using a multi-mode microplate reader (Synergy 2 SIAFRT, BioTek, Winooski, USA). To avoid the influence on biochemical and hematological parameters caused by changes in plasma volume during physical effort, an appropriate hematocrit converter formula was used^27,34^.

### Statistical analysis

Normal distribution was examined using the Shapiro–Wilk test. Differences between T_1_, T_2_, and T_3_ were analyzed using repeated ANOVA measures. Relative reliability was assessed using the intraclass correlation coefficient (ICC) between T_1_, T_2_, and T_3_. The ICC gives the ratio of variances due to differences between subjects. ICC < 0.40 was considered low, between 0.40 and 0.70 acceptable, between 0.70 and 0.90 good, and > 0.90 excellent. However, ICC does not give an indication of the accuracy of individual measurements. Absolute relativity was calculated as standard error of measurement (SEM), which quantifies the precision of the individual measurements. The usefulness of the test was assessed by calculating the smallest worthwhile change (SWC). The ability of the test to detect small and meaningful changes was rated as good if SEM ≤ SWC, satisfactory when SEM = SWC, and marginal in cases with SEM ≥ SWC. Minimal Detectable Change (MDC), which is the minimal amount of change that a measurement must show to be greater than the within subject variability and measurement error, also referred to as the sensitivity to change, was also calculated. Associations between the FGB score and aerobic capacity were measured using the Pearson correlation coefficient. The following criteria were adopted for the interpretation of the magnitude of the correlation: trivial (*r* < 0.1), small (0.1 ≤ *r* < 0.3), moderate (0.3 ≤ *r* < 0.5), large (0.5 ≤ *r* < 0.7), very large (0.7 ≤ *r* < 0.9), nearly perfect (0.9 ≤ *r* < 1), and perfect (*r* = 1). The agreement of two methods was evaluated using the Bland–Altman method after data normalization^36^. Normalization was done by subtracting the mean and dividing by standard deviation.

### Ethics approval

All procedures performed were in accordance with the ethical standards of the institutional and national research committee (Bioethics Committee at Poznan University of Medical Sciences, Poznan, Poland (Decision no. 173/15 of 5 February 2015)) and with the 1975 Helsinki declaration and its later amendments or comparable ethical standards.

### Consent to participate

All participants signed an informed consent.

## Practical applications

This work proposes the first evaluation of the reliability and validation of a specific test to measure HIFT performance. Our study indicated that FGB is a reliable test that can be used in order to measure changes in cross-training performance caused by an intervention. We also showed that cross-training performance is correlated to aerobic fitness, which gives more insight into the physiology of the test. It shows that aerobic fitness, even though underestimated by most of the cross-training athletes, can be an important contributor to success. Our findings could serve as guidance for scientists, as well as coaches and athletes who consider achieving their scientific and/or training goals based on the cross-training specific Fight Gone Bad workout.

## Data Availability

The datasets used and/or analyzed during the current study are available from the corresponding author on request.
